# A Novel Dipeptide NGF Mimetic GK-2 Selectively Activating the PI3K/AKT Signaling Pathway Promotes the Survival of Pancreatic β-Cells in a Rat Model of Diabetes

**Published:** 2019

**Authors:** R. U. Ostrovskaya, S. V. Ivanov, T. A. Gudasheva, S. B. Seredenin

**Affiliations:** V. V. Zakusov Research Institute of Pharmacology, Baltiyskaya Str. 8, 125315, Moscow, Russia

**Keywords:** diabetes, GK-2, metformin, NGF, neurotrophins, PI3K/Akt pathway

## Abstract

We investigated the cytoprotective effect of a novel low-molecular-weight NGF mimetic, GK-2 (hexamethylenediamide bis-N-monosuccinyl-L-glutamyl-L-lysine), on pancreatic β-cells. The neuroprotective effect of GK-2 had been previously shown to be associated with selective activation of the PI3K/Akt signaling pathway. In this study, rats with streptozotocin (STZ)-induced type 2 diabetes mellitus were used. Metformin was used as a reference drug. STZ was immunohistochemically demonstrated to reduce the number of β-cells and affect their morphological structure. Treatment of diabetic animals with GK-2 (at a dose of 0.5 mg/kg intraperitoneally or 5 mg/kg orally) or metformin (300 mg/kg orally) for 28 days reduced the damaging effect of STZ. The effect of GK-2 on manifestations of STZ-induced diabetes, such as hyperglycemia, weight loss, polyphagia, and polydipsia, was comparable to that of metformin, while the cytoprotective activity of GK-2 was slightly stronger than that of metformin. A strong positive correlation between morphometric parameters and the blood glucose level was revealed. The GK-2 cytoprotective effect on β-cells is supposed to manifest through the PI3K/Akt signaling pathway.

## INTRODUCTION

In their search for new ways to treat diabetes mellitus, the attention of researchers has focused on the similarity between the neurochemical mechanisms regulating the functions of neurons and pancreatic β-cells. This similarity includes common receptors (in particular, GABA [1], serotonin [2], and glutamate [3] receptors), as well as similar enzymes (e.g., glycogen synthase kinase 3 (GSK-3) [4]) and transcription factors (in particular, HIF [5, 6]). An additional argument in favor of neuron and β-cell similarity is the fact that β-cells, during their development, effect neuron-like processes [7] and that the neurons of the developing brain contain insulin (a pancreatic β-cell hormone), glucagon (a pancreatic α-cell hormone), and ghrelin (a hormone secreted by gastrointestinal tract cells) [8].

The similarity between the mechanisms regulating the functions of neurons and β-cells is supported by the involvement of the neurotrophic factors NGF [7] and BDNF [9] in the growth and differentiation of these cells. Along with the misfolding of proteins (β-amyloid in neurons, amyloid polypeptide in pancreatic islets), oxidative stress, and insulin resistance [10], neurotrophin dysfunctions [11] are common to Alzheimer’s disease (AD) and type 2 diabetes mellitus (T2DM).

Pancreatic β-cells were found to secrete a biologically active NGF, with its secretion being enhanced by glucose. Even short-term exposure to NGF dramatically increases glucose-stimulated insulin secretion. Insulin secretion drops in the presence of monoclonal NGF antibodies [12]. Along with this, compound K252a, a specific TrkA inhibitor [13], inhibits increased insulin secretion. It is important to emphasize that TrkA receptors are found only in β-cells but not in the α-cells of the pancreas [14].

By using a primary culture of human pancreatic islets, Peerucci *et al*. [15] not only confirmed that human β-cells, like rat ones, express the NGF but also showed that inhibition of this neurotrophin by monoclonal anti- NGF antibodies enhances β-cell apoptosis. Increased apoptosis of β-cells in the case of NGF deficiency was found to be associated with a decreased activity of PI3K/Akt, one of the main TrkA signaling pathways [15].

Attempts to create drugs that are effective, in particular in neurodegenerative diseases, have been underway since the discovery of NGF [16]. However, pharmacokinetic limitations related to the properties of a full-length NGF molecule, such as low biological stability, inability to penetrate biological barriers upon systemic administration, and side effects, have prevented the use of NGF for replacement therapy. Several companies and laboratories have searched for neurotrophin mimetics [17], but there have been no reports of NGF mimetics with satisfactory pharmacokinetic properties.

A working hypothesis positing that certain neurotrophins interacting with the same receptor may activate different signaling pathways, causing different neurotrophins effects, was formulated at the Department of Pharmaceutical Chemistry of the Zakusov Research Institute of Pharmacology [18]. This hypothesis provided a basis for a new direction in pharmacological research for the development of effective low-molecular-weight neurotrophin mimetics free of side effects. According to the proposed hypothesis, a β-turn Asp^93^-Glu^94^-Lys^95^-Gln^96^ of the 4^th^ NGF loop, which is the most exposed to the outside and, therefore, may play a major role in the interaction between the NGF and the receptor, was used to create a dipeptide mimetic called GK-2 (Patent of the Russian Federation No. 2410392, 2010; Patent US 9,683,014 B2, 2017; Patent CN 102365294 B, 2016) [19]. The compound comprises a central dipeptide fragment Glu^94^-Lys^95^ that, according to stereochemical principles, may exhibit the most deep penetration into the receptor-binding site and be the most fully recognized by the receptor. The peripheral Asp^93^ residue was substituted by its bioisostere, a succinic acid moiety, and the Gln^96^ residue was substituted by an amide group. The purpose of these substitutions was to stabilize the β-turn conformation and increase the resistance of the compound to peptidases. The NGF is a homodimer; therefore, the linking of two β-turn mimetics with a hexamethylenediamine spacer provided a dimeric dipeptide, hexamethylenediamine *bis*-(monosuccinyl-glutamyl-lysine).

The main activities of NGF are known to be effected through its interaction with the TrkA receptor tyrosine kinase; wherein, signal transduction associated with TrkA activation mainly involves the phosphatidylinositol 3-kinase (PI3K/Akt) and mitogen-activated protein kinase (MAPK/Erk) signaling pathways, with the first being associated with the regulation of survival [20], and the second being mainly associated with morphological differentiation.

The neuroprotective activity of GK-2 was studied *in vitro *on both immortalized and primary cell cultures. Micromolar and nanomolar GK-2 concentrations were shown to increase the cell survival impaired by exposure to hydrogen peroxide, glutamic acid, and 1-methyl-4-phenyl-1,2,3,6-tetrahydropyridine (MPTP) [21]. Experiments on HT-22 cells using the Western blot analysis with antibodies to phosphorylated and non-phosphorylated Akt and Erk kinases demonstrated that GK-2 activated Akt within the same time intervals as full-length NGF did (after 15, 30, 60, and 180 min). Under similar conditions, GK-2, unlike NGF, did not cause increased phosphorylation of Erk kinases. The effects of the selective inhibitors of phosphatidylinositol 3-kinase and MAPK kinase LY294002 and PD98059, respectively, was studied to clarify the role played by various signaling pathways in the neuroprotective activity of GK-2. Incubation of HT-22 cells with LY294002 (100 μM) or PD98059 (50 μM) demonstrated that the neuroprotective effects of both NGF and GK-2 were completely inhibited by LY294002, but not by PD98059. These data suggest that implementation of the neuroprotective effects of GK-2 is associated with activation of the PI3K/Akt pathway, but not with the MAPK/Erk signaling pathway [22].

*In vivo *experiments provided convincing evidence of the GK-2 neuroprotective effects. The efficacy of GK-2 was demonstrated in various models of Alzheimer’s disease (septo-hippocampal transection, cholinergic deficiency caused by prolonged administration of scopolamine, and on a model induced by administration of streptozotocin into the cerebral ventricles [23]), focal and global cerebral ischemia [24], and hemorrhagic stroke [25].

In recent years, we have developed the concept of a potential antidiabetic effect of neuroprotective agents [26, 27]. Based on this concept, we studied compound GK-2 on a model of streptozotocin (STZ)-induced type 2 diabetes mellitus. We demonstrated that systemic administration of GK-2 eliminates the hyperglycemic effect of the diabetogenic toxin [Patent of the Russian Federation 2613314, 2017] and reduces the behavioral disorders associated with diabetes in mice [28].

This study was performed to determine whether the NGF mimetic GK-2, which exhibits pronounced neuroprotective activity, could also protect β-cells. It was reasonable to compare the cytoprotective effect of GK-2 with that of metformin not only because metformin is an antidiabetic drug of the first choice [29], but also because metformin, like GK-2, activates the PI3K/Akt signaling pathway [30]. The study’s ob jective was also to compare the effects of GK-2 and metformin on T2DM manifestations, such as hyperglycemia, weight loss, polydipsia, and polyphagia. It was important to evaluate the extent to which the cytoprotective and antihyperglycemic effects of these compounds correlate with each other.

## EXPERIMENTAL

**Animals**

The experiments were performed on adult Wistar male rats with an initial body weight of 250–270 g, which were received from the Stolbovaya Central Laboratory for Animal Breeding (Moscow Region, Russia). The animals had free access to feed (except for 16 hours before STZ administration) and to drinking water. The animals were kept in accordance with SP 2.2.1.3218-14 No. 51 of August 29, 2014. The experiments were approved by the Committee for Biomedical Ethics of the Zakusov Research Institute of Pharmacology.

**Compounds**

STZ (Sigma, USA) was used as a diabetogenic toxin. The NGF mimetic GK-2 and metformin (Siofor, Berlin-Chemie Menarini, Germany) dissolved in saline solution (SS) were used.

**Experiment design**

T2DM was induced by a single intraperitoneal injection of a freshly prepared STZ solution at a dose of 45 mg/kg dissolved in cold citrate buffer (pH 4.5). The choice of this dose for the induction of T2DM was associated with a previously detected decrease in the blood insulin level by 48% and preservation of 30% of viable β-cells in the pancreas [27]. The glucose level in blood sampled from the tail vein was determined using a One Touch Ultra device (USA). The first measurement was performed 72 h after administration of STZ. Only animals with a blood glucose level of at least 15 mmol/L were included further in the experiment. The experiment included two series. In the first series, rats (n = 48) were randomly divided into four groups: 1) rats of the passive control group (n = 12) received a single intraperitoneal injection of citrate buffer on day 1 of the experiment and then 2 mL/kg of SS for the next 28 days; 2) rats of the active control group (n = 12) were administered 45 mg/kg of STZ on day 1 of the experiment, followed by administration of SS for the next 28 days; 3) rats of the first experimental group (n = 12) received a single intraperitoneal injection of 45 mg/kg STZ, followed by intraperitoneal injections of GK-2 at a dose of 0.5 mg/kg for the next 28 day; 4) rats of the second experimental group (n = 12) received a STZ injection, followed by oral administration of GK-2 at a dose of 5 mg/kg for 28 days (a tenfold increase in the dose when switching from intraperitoneal to oral administration is used for most dipeptide drugs) [31, 32]. In the second series of the experiment (n = 36), rats were divided into three groups: the passive control group (n = 12); the active control group (n = 12) (with a design similar to that of the first series); the experimental group (n = 12) that received a single injection of STZ at a dose of 45 mg/ kg, followed by oral administration of metformin at a dose of 300 mg/kg (the most common dose used in the experiment) for the next 28 days. The glucose level in all groups of both series was determined at 1, 7, 14, 21, and 28 days of drug administration. Food and water consumption were measured daily; the animals were weighed every 3 days. The animals were euthanized by decapitation, and then the pancreatic islet was immunohistochemically analyzed.

**Sample preparation**

The extracted pancreases of rats from the experimental groups were fixed in 10% neutral formalin (pH 7.4) (Sigma, USA). The samples were dehydrated in an ascending series of alcohols and xylene and immersed in paraffin blocks. Sections 5 μm thick were prepared using a microtome (Jung RM2035, Germany). Slides (4 to 5 sections per glass) were kept in a dry bath at 40°C for 60 min. Before treatment with antibodies, the slides were washed out twice in xylene, hydrated in a descending series of alcohols, and washed in phosphate buffer (PBS, Sigma, USA) for 10 min. The slides were treated with a 3% hydrogen peroxide solution for 10 min to neutralize endogenous peroxidases.

**Immunohistochemical reaction**

The slides were incubated with a 10% solution of normal goat serum (Abcam, UK) at room temperature for 1 h to prevent nonspecific staining due to binding of primary antibodies to tissue components. Sections were treated with primary monoclonal anti-insulin antibodies (anti-insulin GP 1:500, Abcam, UK) in phosphate buffer. The treated slides were left in a wet chamber at temperatures of 2–4°C for 24 h.

To visualize the immunohistochemical reaction results, the slides were incubated with secondary monoclonal peroxidase-labeled antibodies (anti-GP Rabbit 1:500, Abcam, UK) at room temperature for 1 h, followed by treatment with a reagent kit (DAB Vector Peroxidase, USA). The ready slides were again dehydrated in an ascending series of alcohols and xylene and immersed in a Eukitt medium (Panreac, Spain). The reliability of the study results was achieved using negative antigen and antibody controls.

**Microscopic analysis**

A morphometric analysis was performed using an Aristoplan microscope (Leitz, Germany) equipped with a DCM-800 digital camera (Micromed, Russia), a personal computer, and the ScopePhoto software, at a magnification of ×400 (to calculate the total area of the slides) and ×1,600 (to calculate the area of islets and β-cells). The proportion of β-cells in the total slide area, the number of pancreatic islets, and the mean islet size were calculated.

Given the published data on a heterogeneous response of pancreatic islets of various sizes to the damaging effects of STZ [33], we performed a differential analysis of the islet area and calculated the percentage of islets of each size range (less than 500 μm2, 501–2,500 μm2, 2,501–10,000 μm2, and more than 10,001 μm2).

**Statistical analysis**

Statistical data processing was performed using the Biostat software. The distribution of data was characterized using the Shapiro-Wilk test. Due to the normal data distribution, the statistical significance of differences among groups was assessed by the ANOVA test. The mean value M and the standard error of the mean SEM were calculated. The difference of means was considered statistically significant at *p * < 0.05.

To comparatively characterize the dynamics and strength of the drug effect, a relative antihyperglycemic activity indicator (*Ar*) was calculated using the formula:

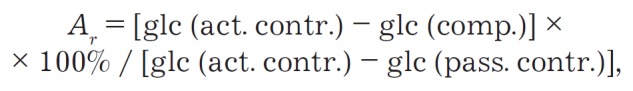



where glc (act. contr.) is the plasma glucose level in the STZ/SS group; glc (comp.) is the glucose level in the STZ/GK-2 or STZ/metformin groups; and glc (pass. contr.) is the glucose level in animals injected with SS.

To compare the cytoprotective activity of GK-2 and metformin, a relative indicator (Cpa) was calculated using the formula:

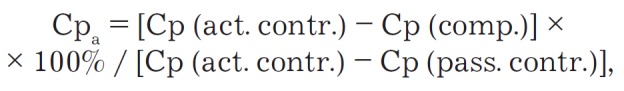



where Cp (act. contr.) is the percentage of β-cells in the cross section of the pancreases of rats from the STZ/SS group; Cp (comp.) is the percentage of β-cells in the cross section of the pancreases of rats from the STZ/GK-2 or STZ/metformin group; and Cp (pass. contr.) is the percentage of β-cells in the cross section of the pancreases of rats injected with SS.

## RESULTS

As follows from the data presented in *Table 1*, the blood glucose level in the animals of the passive control group was 5–6 mmol/L during the entire observation period, while in rats with DM it increased more than 3-fold on day 7 of the experiment. At days 14, 21, and 28, the glucose level in the active control animals remained consistently high (more than 20 mmol/L). Pronounced decompensation of DM in the active control group was also evidenced by weight loss, polyphagia, and polydipsia. Pronounced antidiabetic activity of GK-2 was revealed. By the end of the first week of GK-2 i/p administration, a significant decrease in the glycemic level was observed. Orally administered GK-2 retained its activity. Metformin also reduced the glycemic level (*Table 2*). According to the relative indicator *A*r (*Table 3*), the effect of metformin developed later than that of GK-2; however, on the 3^rd^ and 4^th^ weeks, the *Ar *indicator of metformin was slightly higher than that of GK-2.

**Table 1:** The effect of GK-2 on the basal glucose level in rats of series 1

	Group	Basal glucose level (mmol/L; M ± m)				
		Day 1	Day 7	Day 14	Day 21	Day 28
1	Passive control	6.0±0.3	5.9±0.2	6.4±0.2	6.2±0.2	6.4±0.2
2	Active control	21.0±1.5^**^	30.0±1.2^**^	23.6±1.9^**^	23.4±2.1^**^	24.5±1.9^**^
3	Intraperitoneal GK-2	23.1±1.5	19.1±2.9^*^	14.3±2.1^*^	12.5±1.4^*^	10,8±0.9^*^
4	Oral GK-2	21.9±2.4	25.2±2.4^*#^	13.5±1.5^*^	13.2±2.1^*^	12.2±1.4^*^

^*^– Statistical significance of differences between the experimental group and active control, p < 0.05.

^**^– Statistical significance between passive control and active control, p < 0.05.

^#^– Statistical significance between experimental groups, p < 0.05.

A favorable effect of both drugs was also confirmed by changes in body weight. While healthy rats gained weight over the experimental period (+16.2% of the baseline value), STZ caused a significant decrease in the body weight (–10.3%). GK-2 and metformin therapy attenuated the STZ-induced weight loss (the difference between the initial and final values was 1.6 and –0.7% for i/p and oral GK-2 administration, respectively, and –1.3% for metformin) (differences from active control animals were statistically significant, *p * < 0.01).

**Table 2:** The effect of metformin on the basal glucose level in rats of series 2

	Group	Basal glucose level (mmol/L; M ± m)				
		Day 1	Day 7	Day 14	Day 21	Day 28
1	Passive control	5.4±0.3	5.6±0.2	5.8±0.4	5.8±0.2	5.9±0.3
2	Active control	17.6±0.9^**^	20.5±1.8^**^	22.2±1.2^**^	25.6±2.0^**^	29.3±1.7^**^
3	Metformin, 300 mg/kg	18.1±0.7	21.3±2.8	15.6±2.2^*^	8.8±1.0^*^	11.0±1.6^*^

^*^– Statistical significance of differences between the experimental group and active control, p < 0.05.

^**^– Statistical significance of differences between passive control and active control, p < 0.05.

**Fig. 1:**
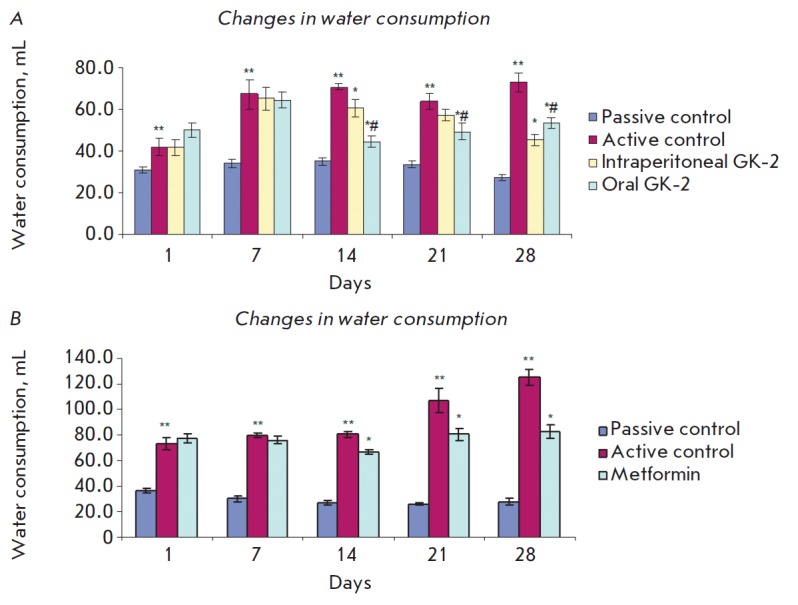
The influence of GK-2 and metformin on water consumption in Wistar rats. *A *– GK-2; *B *– metformin. * – Statistical significance of differences between the experimental group and active control, *p * < 0.05. ** – Statistical significance of differences between passive control and active control, *p * < 0.05. # – Statistical significance of differences between experimental groups, *p* < 0.05

The antidiabetic activity of GK-2 is confirmed by a decrease in polydipsia, which is the important DM indicator. While the animals from the active control group had a marked thirst (daily water consumption in untreated diabetic rats was 450% higher than that in healthy rats), diabetic animals treated with GK-2 consumed 62% and 27% less water in the case of i/p and oral administration, respectively, while metformin-treated rats consumed 33% less water than animals of the active control group (differences from active control values were significant, *p* < 0.01) (*Fig. 1*).

GK-2 and metformin also affected polyphagia. While, by the end of the experiment, animals of the active control group consumed 18.3% more feed than healthy rats, this difference in the GK-2 group amounted to 2.0 and 8.5% (i/p and oral administration, respectively) (differences from active control indicators were statistically significant, *p * < 0.01). For metformin, this difference was less pronounced and amounted to 15.0%.

**Table 3:** Relative indicators of antihyperglycemic activity (Ar) of GK-2 and metformin

	Compound	Ag (%)				
		Day 1	Day 7	Day 14	Day 21	Day 28
1	Intraperitoneal GK-2	–13.5	45.2	54	63.3	75.4
2	Oral GK-2	–6	19.9	59.1	59.2	68.2
3	Metformin	–4.2	–5.7	40.3	84.8	78.2

The cytoprotective activity of both compounds was evaluated by immunohistochemical analysis, which is highly specific for Langerhans β-cells. The results of a morphometric analysis of pancreatic sections are given in *Table 4* and *Table 5*.

**Table 4:** The effect of GK-2 on the morphometric parameters of rat pancreatic islets

	Group	Total β-cell area		Mean islet area, μm^2^	Number of islets
		absolute, μm^2^	% of the islet area		
1	Passive control	259,925±46,353	19.9±2.3	20,088±3,920	13±2
2	Active control	79,131±21,266^**^	8.3±2.1^**^	5,983±1,805^**^	14±3
3	Intraperitoneal GK-2	180,076±36,026^*^	13.3±1.7^*^	15,567±3,820^*^	12±3
4	Oral GK-2	175,907±31,357	14.0±2.0^*^	15,167±1,895^*^	12±2

^*^– Statistical significance of differences between the experimental group and active control, p < 0.05.

^**^– Statistical significance of differences between passive and active control, p < 0.05.

**Fig. 2:**
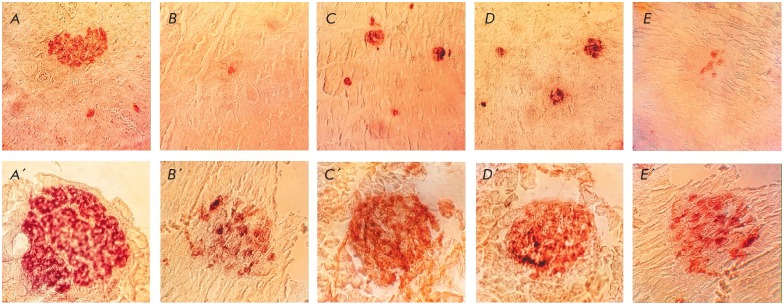
Pancreatic islets of animals from different groups. Magnification ×620 (upper panel) and ×1,600 (bottom panel). *A*, *A’ *– passive control animals; *B*, *B’ *– active control animals; *C*, *C’ *– diabetic rats treated with intraperitoneal GK-2; *D*, *D*’ – diabetic rats treated with oral GK-2; *E*, *E’ *– diabetic rats treated with metformin

The obtained data indicate a decrease in the number of islets, as well as in the absolute and relative numbers of β-cells in the group of untreated diabetic animals. The islets of rats in this group are characterized by changed shapes and the presence of dystrophic elements (*Fig. 2B*). Treatment of diabetic rats with GK-2 and metformin for 28 days leads to noticeable restoration in the proportion of β-cells and their morphological characteristics (*Fig. 2 C, D, E*). The relative cytoprotective activity of GK-2 is slightly higher than that of metformin: CP is 49.5 and 43.4 for GK-2 upon i/p and oral administration, respectively, and 30.3 for metformin.

**Table 5:** The effect of metformin on the morphometric parameters of rat pancreatic islets

	Group	Total β-cell area		Mean islet area, μm^2^	Number of islets
		absolute, μm^2^	% of the islet area		
1	Passive control	225,270±17,005	20.0±1.3	18,477±2,142	12±1
2	Active control	70,181±20,313^**^	5.8±2.1^**^	7,646±1,654^**^	9±2
3	Metformin	115,353±23,845	10.1±0.8^*^	9,749±1,714	12±2

^*^– Statistical significance of differences between the experimental group and active control, p < 0.05.

^**^– Statistical significance of differences between passive and active control, p < 0.05.

**Fig. 3:**
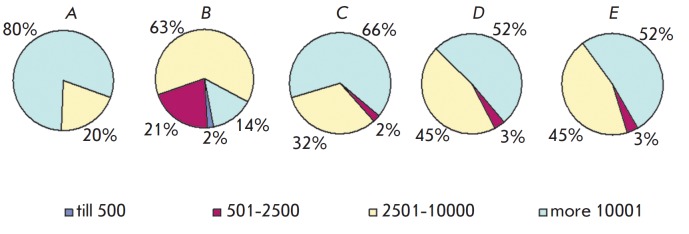
Percentage (%) of β-cell islets of different areas (μm2). *A *– passive control animals; *B *– active control animals; *C *– diabetic rats treated with intraperitoneal GK-2; *D *– diabetic rats treated with oral GK-2; *E *– diabetic rats treated with metformin

The results of a differential assessment of the β-cell islet area are of particular interest (*Fig. 3*). Large islets (2,501–10,000 μm2 and more than 10,001 μm2) prevailed in the group of healthy animals; in untreated diabetic animals, the number of large islets sharply decreased and the number of small ones (501–2,500 μm2 and 2,501–10,000 μm2) increased. The use of GK-2 and metformin led to an increase in the proportion of large islets.

**Fig. 4:**
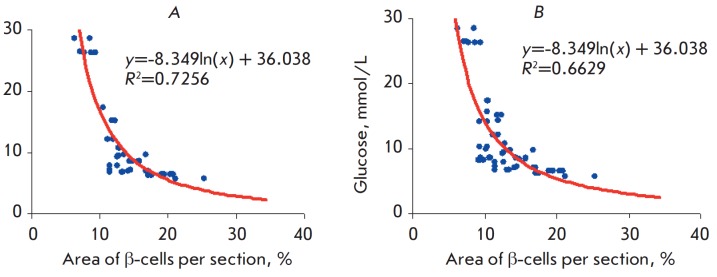
Correlation between the blood glucose level and the relative β-cell area in pancreatic sections. *A *– GK-2; *B *– metformin

The area of β-cells (percentage of the total pancreatic islet area), which characterizes the degree of pancreas damage upon treatment with GK-2 and metformin, correlates with the blood glucose level (the correlation coefficients were 0.7256 and 0.6629, respectively) (*Fig. 4*).

## DISCUSSION

An important trend in modern diabetology is the search for ways to protect β-cells from the damaging effects of metabolic changes characteristic of T2DM: lipoglucotoxicity, oxidative stress, DNA alkylation, ATP deficiency, and reduced levels of mature neurotrophins [34, 35]. In the review *Regulating the beta cell mass as a strategy for type-2 diabetes treatment *[36], L. Song and co-authors indicate that modern antidiabetic therapy, which is mainly aimed at increasing the secretion and efficacy of insulin as well as at glucose uptake, is symptomatic. It is emphasized that etiotropic (disease-modifying) therapy should be based on the use of substances that prevent the loss of β-cells, increasing their survival rate without toxic effects on other organs.

These substances could include a wide group of antioxidant compounds that neutralize free radicals, thereby protecting pancreatic β-cells from death. However, a number of researchers indicate the risk of overestimating the efficacy of antioxidants in DM, which are able only to absorb existing radicals but are not able to prevent the generation of new ones [37]. Compounds of other classes, such as histone deacetylase and GSK- 3β inhibitors, may be used. However, these molecules are involved in the regulation of a variety of processes; therefore, there is a risk of undesirable reactions, including a pro-oncogenic effect. The most convincing evidence of this effect was obtained for dipeptidyl peptidase-4 (DPP-4) inhibitors [38].

Neurotrophins that are able to increase survival by reducing apoptosis not only in neuronal, but also in nonneuronal systems might be promising agents for T2DM, but they possess unfavorable pharmacokinetic properties. An original approach to the development of systemically active low-molecular-weight NGF and BDNF mimetics free of the disadvantages of a full-length molecule, which was developed at the Zakusov Research Institute of Pharmacology, has led to the creation of a series of compounds (Patent RF No. 2410392, 2010; Patent US 9,683,014 B2, 2017; Patent CN 102365294 B, 2016). One of them is compound GK- 2, a NGF loop 4 mimetic with a wide spectrum of *in vivo *and *in vitro *neuroprotective activities.

By using a highly sensitive and selective immunohistochemical analysis, we revealed for the first time the cytoprotective effect of compound GK-2 on pancreatic β-cells. While STZ at a diabetogenic dose reduces the number of Langerhans islets, reduces the absolute and relative numbers of β-cells, changes their shape, and induces the emergence of dystrophic elements, administration of the original dimeric dipeptide NGF mimetic, compound GK-2, to animals with developed diabetes (glycemia above 20 mmol/L) significantly reduces the severity of these morphological changes. A differential analysis of the islet size showed large islets (with an area of 2,501–10,000 μm2 and more than 10,001 μm2) predominant in healthy animals, small islets (up to 500 μm2) prevailing in the untreated STZ group, while the use of GK-2 increased the number of large islets in diabetic rats. It is interesting to correlate this fact with the ideas [33] that this increase indicates an attenuation of apoptosis. The degree of morphological changes, which is evaluated based on the ratio of the β-cell area to the total pancreatic islet area, clearly correlates with the antihyperglycemic effect of GK-2.

The glucose transporter GLUT2 has been repeatedly reported to enable selective accumulation of STZ in β-cells, which causes their apoptosis [39, 40]. STZ-induced apoptosis is associated with oxidative stress and a shift in the ratio of the NGF precursor and mature NGF towards a predominance of the precursor with a characteristic pro-apoptotic effect [41]. Earlier, GK-2, like the full-length NGF molecule, was shown to increase the survival of neuronal cells exposed to hydrogen peroxide, glutamic acid, and MPTP [21]. Also, the effect of neurotrophin mimetics, which affect different translational pathways, on manifestations of STZ-induced diabetes was studied. Only mimetics activating the PI3K/Akt pathway (the NGF loop 4 mimetic GK-2 and the BDNF loop 1 mimetic GSB-214) exhibited an antihyperglycemic effect, while the BDNF loop 2 mimetic activating the MAPK/Erk pathway (GTS-201) lacked antihyperglycemic activity [42].

Dysfunction of β-cells in T2DM is known to be related to a decreased activity of the PI3K/Akt pathway [43], whose role as a factor controlling maintenance of the β-cell volume and function has been shown *in vivo *and *in vitro *[44]. Genetically modified mice with PI3K/Akt pathway deficiency develop severe diabetes in the setting of enhanced β-cell apoptosis [45, 46]. The PI3K/Akt pathway deficiency characteristic of diabetes is reproduced in a STZ-induced diabetes model [47]. It is important to emphasize that transgenic mice overexpressing a constitutively active form of this pathway are characterized by an increased size and number of pancreatic β-cells, as well as by elevated tolerance to a glucose load [48].

The data on the role of PI3K/Akt pathway deficiency in the development of β-cell apoptosis in DM, along with the data on the involvement of this pathway in the implementation of the antihyperglycemic effect of GK-2 [42], suggest that the protective effect of GK-2 is also associated with the ability of this NGF mimetic to activate the abovementioned pathway. The fact that GK-2 exerts a cytoprotective effect not only on neurons, as shown earlier [49], but also on β-cells is an important argument in favor of the idea of a similarity between the mechanisms underlying the protection of neurons and β-cells. This is the fundamental aspect of our work. The ability of this systemically active NGF mimetic to protect pancreatic β-cells is of practical importance, because this compound is currently being investigated as a treatment for strokes, and the “coexistence” of stroke and diabetes is well established [50]. The use of GK-2 for combined vascular and diabetic pathology may be associated with long-term administration of the drug; therefore, it is important that both the neuroprotective and cytoprotective GK-2 activities towards pancreatic β-cells are retained upon long-term oral administration.

Metformin is a first-line drug for T2DM and has been successfully used by millions of people worldwide for over 50 years [51]. The mechanism of antidiabetic action of metformin is multicomponent [52]. The most important component of this mechanism is obviously the activation of 5’-AMP-activated protein kinase (AMPK) [53], which leads to the suppression of key gluconeogenesis enzymes in the liver. Metformin increases glucose utilization by the muscles and enhances anaerobic glycolysis in the small intestine. Also, metformin was shown to enhance the expression of NGF and BDNF in a culture of Schwann cells [54]. The effect of metformin was found to be inhibited by a selective inhibitor of the translational PI3K/Akt pathway [30]. The similarity between one of the mechanisms underlying the metformin and GK-2 action, whose effects depend on PI3K/Akt, was the reason for our comparative study of their effects in a STZ-induced T2DM model. High activity of GK-2, which is comparable to that of metformin, defines the practical value of this study.

## CONCLUSION

In this study, the antidiabetic effect of an original systemically active NGF mimetic, GK-2 (hexamethylenediamine- bis-(N-monosuccinyl-L-glutamyl-L-lysine)), previously detected in mice, was reproduced in experiments on rats [28]. Direct evidence of the cytoprotective effect of GK-2 on pancreatic β-cells was, for the first time, obtained in a STZ-induced T2DM model. Cytoprotection of β-cells is a new actual direction attracting the attention of diabetes researchers. Because the protective effect of GK-2 on neurons subjected to different damaging factors was described earlier, the present findings on the attenuation of STZ-induced apoptosis of β-cells during GK-2 therapy represent additional confirmation of the similarity between the neurochemical mechanisms regulating the functions of neurons and pancreatic β-cells and their mechanisms of protection. An important scientific and practical result of this work is the comparability of the effects of GK-2 and metformin, a first-choice standard antidiabetic drug, on the functional manifestations of T2DM (hyperglycemia, weight loss, polyphagia, polydipsia); the cytoprotective effect of GK-2 slightly exceeds that of metformin.

## Abbreviations

T2DM: type 2 diabetes mellitus
AD: Alzheimer’s disease
STZ: streptozotocin
NGF: nerve growth factor
TrkA: tropomyosin receptor kinase A
